# Renal artery embolization for spontaneous hemorrhage in patients with acquired cystic kidney disease: A 20-year single-center experience

**DOI:** 10.12669/pjms.37.4.3999

**Published:** 2021

**Authors:** Cheng Shi Chen, Hyemin Ahn, Ji Hoon Shin, Hai-Liang Li, Jong Woo Kim, Alrashidi Ibrahim, Hee Ho Chu

**Affiliations:** 1 Cheng Shi Chen, MD. Department of Radiology, The Affiliated Cancer Hospital of Zhengzhou University, Zhengzhou, China; 2 Hyemin Ahn, MD. Department of Radiology and Research Institute of Radiology, Asan Medical Center, Seoul, Korea; 3 Ji Hoon Shin, MD. Department of Radiology, The Affiliated Cancer Hospital of Zhengzhou University, Zhengzhou, China Department of Radiology and Research Institute of Radiology, Asan Medical Center, Seoul, Korea; 4 Hai-Liang Li, MD. Department of Radiology, The Affiliated Cancer Hospital of Zhengzhou University, Zhengzhou, China; 5 Jong Woo Kim, MD. Department of Radiology and Research Institute of Radiology, Asan Medical Center, Seoul, Korea; 6 Alrashidi Ibrahim, MD. Department of Radiology, Prince Sultan Military Medical City, Riyadh, Saudi Arabia; 7 Hee Ho Chu, MD. Department of Radiology and Research Institute of Radiology, Asan Medical Center, Seoul, Korea

**Keywords:** Acquired cystic kidney disease, Contrast-induced nephropathy, Spontaneous hemorrhage, Renal artery embolization

## Abstract

**Objectives:**

To evaluate the safety and effectiveness of transcatheter arterial embolization for controlling spontaneous hemorrhage in patients with acquired cystic kidney disease (ACKD).

**Methods:**

This retrospective study included 18 patients with ACKD (M:F=13:5; mean age, 56 years) who underwent renal artery embolization to control spontaneous hemorrhage between January 2001 and September 2020. The underlying etiology and clinical presentations were reviewed and previous computed tomography (CT) findings were analyzed. Furthermore, angiographic and embolization details, technical and clinical successes, and complications were assessed.

**Results:**

Subcapsular, perirenal, and pararenal hematomas were observed on CT scans for all patients. Contrast extravasation was observed in 15 / 17 patients (88%) on contrast-enhanced CT scans. Angiography showed active bleeding in 14 patients (78%; contrast extravasation [n=6], pseudoaneurysm [n=3], and both [n=5]), suspicious bleeding in 1 (5%), and no bleeding in 3 (17%). The technical and clinical success rates were 100% and 94% (17/18), respectively. Total and partial embolization was performed in 14 (78%) and 4 (22%) cases, respectively. Subsequent surgical nephrectomy was required for one patient with clinical failure due to recurrent bleeding despite total embolization. Procedure-related complications included mild post-embolization syndrome in one patient and contrast-induced nephropathy in five patients (28%) without long-term complications.

**Conclusions:**

Renal artery embolization is safe and effective for controlling spontaneous renal hemorrhage in patients with ACKD.

## INTRODUCTION

Acquired cystic kidney disease (ACKD) refers to multiple cysts occurring in the bilateral kidneys of patients with chronic kidney disease without a hereditary cause and is known to be associated with the duration of dialysis.^1^ Bleeding in patients with ACKD is a well-known complication, and approximately 50% of these patients develop hemorrhagic cysts. The cause of hemorrhagic cyst rupture is thought to be an increase in the intracystic pressure due to recurrent hemorrhage, combined infection, or obstructive uropathy. Anticoagulant use and uremia-associated platelet dysfunction can also be contributing factors.^2^

With the emerging techniques of interventional radiology, transcatheter arterial embolization is considered the first-line therapy for life-threatening arterial renal hemorrhage.^3^ Conservative interventional treatment and imaging follow-up are suggested to avoid unnecessary nephrectomy,^4^ and several cases of successful interventional treatment have been reported.^5^-^7^ However, to date, only case reports have been published, and no studies are specific to patients with ACKD regarding detailed angiographic findings and comprehensive analysis of its efficacy and clinical outcomes. Therefore, the purpose of this retrospective study was to evaluate the effectiveness and safety of renal artery embolization for controlling massive bleeding in patients with ACKD.

## METHODS

This study was approved by the hospital institutional review board (Ref# 2020-0866 dated December 15, 2020). The cases of patients who underwent renal artery embolization between January 2001 and September 2020 were retrospectively analyzed. Patients with chronic kidney disease and spontaneous hemorrhage were selected first, while those with obvious renal tumors or vascular diseases were excluded. A total of 23 patients who had multiple cysts on both kidneys (three or more per kidney) with spontaneous renal hemorrhage were identified. Subsequently, five patients with autosomal dominant polycystic kidney disease were excluded. Finally, 18 patients (13 men and 5 women; mean age, 56 years; range, 32–84 years) were enrolled in this study.

### Angiography and embolization technique

Renal artery embolization was performed by four board-certified interventional radiologists with 15–20 years of clinical experience. The right common femoral artery was accessed; after the right or left renal artery was selected using a 0.035-inch hydrophilic guidewire (Radifocus; Terumo, Tokyo, Japan) and standard 5-F catheter (Cobra catheter; Cook, Bloomington, IN, USA), the renal angiography was performed. A microcatheter was advanced into all identifiable target vessels, which were subsequently embolized.

Embolic agents included polyvinyl alcohol (PVA; Contour; Boston Scientific), n-butyl cyanoacrylate (NBCA; Histoacryl; B. Braun, Melsungen, Germany), microcoils (Tornado or MicroNester; Cook), and gelatin sponge particles (GSP; Gelfoam; Pharmacia & Upjohn, Kalamazoo, Michigan). Subsequently, a final renal arteriography was performed to confirm successful occlusion of the target vessel and no further bleeding.

### Definitions

Angiography findings were classified into (1) active, (2) suspicious, or (3) no bleeding. Active bleeding was defined as contrast extravasation or a pseudoaneurysm. Suspicious bleeding was defined as blurring of the contrast agent without definite active bleeding. Hemodynamic instability was defined as hypotension with a systolic blood pressure less than 90 mmHg despite the use of a vasopressor or the need for a blood transfusion of at least 3 units of packed red blood cells without an adequate hemoglobin increase.^3^,^8^

Technical success was defined as a complete occlusion of the bleeding vessel on immediate post-embolization angiography. Clinical success was defined as the resolution of signs and symptoms of bleeding and no requirement for interventional or surgical hemostasis.

Minor complications were defined as no need for additional treatment or hospitalization overnight for observation. Major complications were defined as the requirement for therapy with minor hospitalization (<48 h), requiring major therapy, prolonged hospitalization (>48 h) or unplanned increase in the level of care, permanent adverse sequelae, or death.^9^ Among the complications, contrast-induced nephropathy (CIN) was defined as a 25% increase in the serum creatinine (sCr) level from baseline within 48–72 h of administration of the contrast agent without another explanatory reason.^10^

## RESULTS

The patients’ clinical characteristics are summarized in Table-I. All patients visited the emergency room for sudden flank or abdominal pain, and six (33%) initially showed hemodynamic instability. The initial estimated glomerular filtration rates of all patients were less than 15 mL/min. A total of 17 patients (94%) were on dialysis for a mean period of 8.8 years (range, 0.8–14 years), and four (22%) had a history of kidney transplantation. One patient (6%) was on conservative treatment for chronic kidney disease.

**Table-I:** Patient characteristics and clinical outcomes.

No./age /sex	CKD cause	Symptom	Dialysis (month)	CT findings	Angiography findings	Embolic agents	Embolization extent	Technical success	Clinical success	F.U. (days)	Remarks
1/69/F	Unknown	Flank pain	168	C.E. (multiple)	C.E., PSA (multiple)	Coil, PVA	Total	Yes	Yes	896	
2/44/M	HTN	Flank pain	9	C.E, PSA (multiple)	PSA (multiple)	GSP, PVA	Total	Yes	Yes	2,559	Ischemic bowel change, CIN
3/51/M	HTN	Flank pain	144	C.E.	Suspicious bleeding	GSP	Total	Yes	Yes	647	PES
4/47/F	MPGN	Flank pain	132	C.E.	C.E.	PVA	Total	Yes	Yes	2,115	
5/54/M	MPGN	Abd. pain	132	C.E.	No bleeding	Coil, PVA	Total	Yes	Yes	2,108	
6/59/F	GN	Flank pain, Hematuria	68	C.E. (multiple)	C.E., PSA (multiple)	NBCA	Total	Yes	Yes	1,960	
7/54/M	DM	Abd. pain	132	C.E.	PSA (multiple)	PVA	Total	Yes	Yes	20	
8/32/F	Unknown	Flank pain	67	C.E.	C.E.	NBCA	Partial	Yes	Yes	1,558	
9/79/F	Unknown	Flank pain	120	C.E. (multiple)	C.E.	PVA	Total	Yes	Yes	732	
10/42/M	DM	Flank pain	96	C.E., PSA (multiple)	C.E., PSA (multiple)	Coil, NBCA	Total	Yes	Yes	703	
11/48/M	DM	Flank pain	21	Hemorrhagic cysts	No bleeding Displaced capsular a,	Coil, PVA	Total, capsular a. embolization	Yes	Yes	560	
12/62/M	HTN	Flank pain	120	Pre-contrast scan only	PSA (multiple)	Coil, GSP	Total	Yes	Yes	493	
13/51/M	MPGN	Flank pain	144	C.E.	C.E.	NBCA	Partial	Yes	Yes	465	CIN
14/43/M	DM	Flank pain	104	C.E.	C.E., PSA (multiple)	Coil, PVA	Total	Yes	No	531	Radical nephrectomy 2 days later
15/71/M	IgAN	Flank pain	136	C.E.	No bleeding	Coil, GSP	Total	Yes	Yes	440	CIN
16/46/M	HTN	Flank pain	72	C.E.	C.E.	GSP, PVA	Partial	Yes	Yes	1,398	CIN
17/64/M	Unknown	Abd. pain	0	Enhancing nodule^¦^	C.E., PSA (multiple)	Coil	Partial	Yes	Yes	18	CIN
18/84/M	Unknown	Flank pain	120	C.E.	C.E.	NBCA, PVA	Total	Yes	Yes	465	

***Abbreviations.*** CKD, chronic kidney disease; HTN, hypertension; MPGN, membranoproliferative glomerulonephritis; GN, glomerulonephritis; DM, diabetes mellitus; IgAN, IgA nephropathy; Abd., abdominal; C.E., contrast extravasation; PSA, pseudoaneurysm; PVA, polyvinyl alcohol; GSP, gelatin sponge particles; NBCA, n-butyl cyanoacrylate; F.U., follow-up; PES, post-embolization syndrome; CIN, contrast-induced nephropathy. § Enhancing nodule was a suspected pseudoaneurysm or renal cell carcinoma.

**Fig.1:**
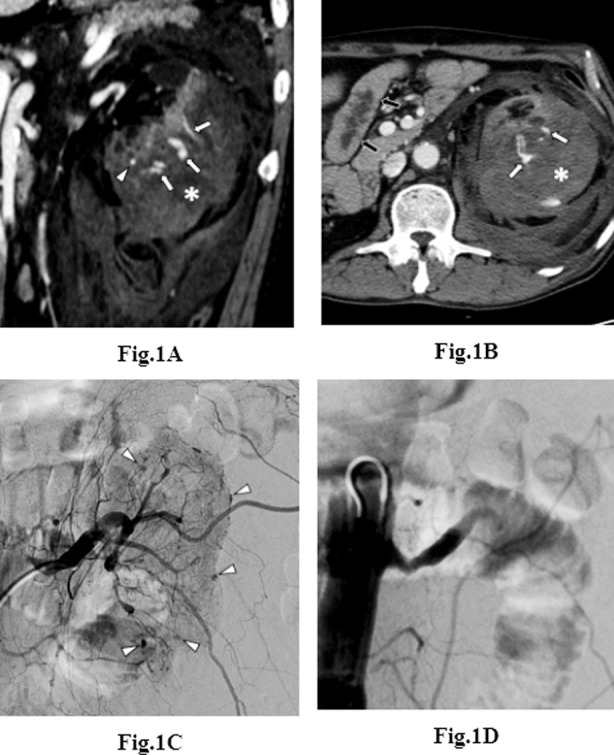
A 44-year-old man (no. 2) with diffuse cortical hemorrhage. a, b Coronal (a) and axial (b) contrast-enhanced computed tomography scans showing multiple foci of contrast extravasation (arrows) and pseudoaneurysm (arrowhead) along the periphery of the renal cortex with a large subcapsular hematoma (asterisks). Note the concentric jejunal wall thickening indicating an ischemic change (black arrows in b). c Left renal angiogram showing multiple pseudoaneurysms (arrowheads) along the periphery of the renal cortex. d Aortogram showing complete occlusion of the left renal artery after embolization with gelatin sponge particles and polyvinyl alcohol.

CT scans were obtained for all 18 patients, with only a pre-contrast CT scan in one patient. Subcapsular, perirenal, and pararenal hematomas were observed in all patients; four of them also showed hemoperitoneum from extension of a retroperitoneal hemorrhage without evidence of active bleeding in the peritoneal organs. Contrast extravasation was observed in 15 / 17 patients (88%) on contrast-enhanced CT scans, and among them, five (29%) showed multiple active bleeding foci along the peripheral renal cortex (Fig.1). One patient (no. 17) showed a nodular-enhancing lesion, which could be a pseudoaneurysm or renal cell carcinoma (RCC). Although RCC was not excluded, interventional treatment was first attempted to maintain the patient’s hemodynamic stability since he was suspected of having ischemic heart disease. The remaining one patient (no. 11) only showed hemorrhagic cysts without evidence of active bleeding. Another combined CT finding was ischemic change to the small bowels like concentric wall thickening due to hypovolemia in one patient (no. 2; Fig.1). This ischemic change was resolved on follow-up CT three weeks later. In another patient (no. 6), a subcapsular hematoma was initially misinterpreted as renal infarction, and intravenous heparinization was initiated, which led to active bleeding with a large retroperitoneal hematoma on a re-scanned CT after 10 h.

**Fig. 2:**
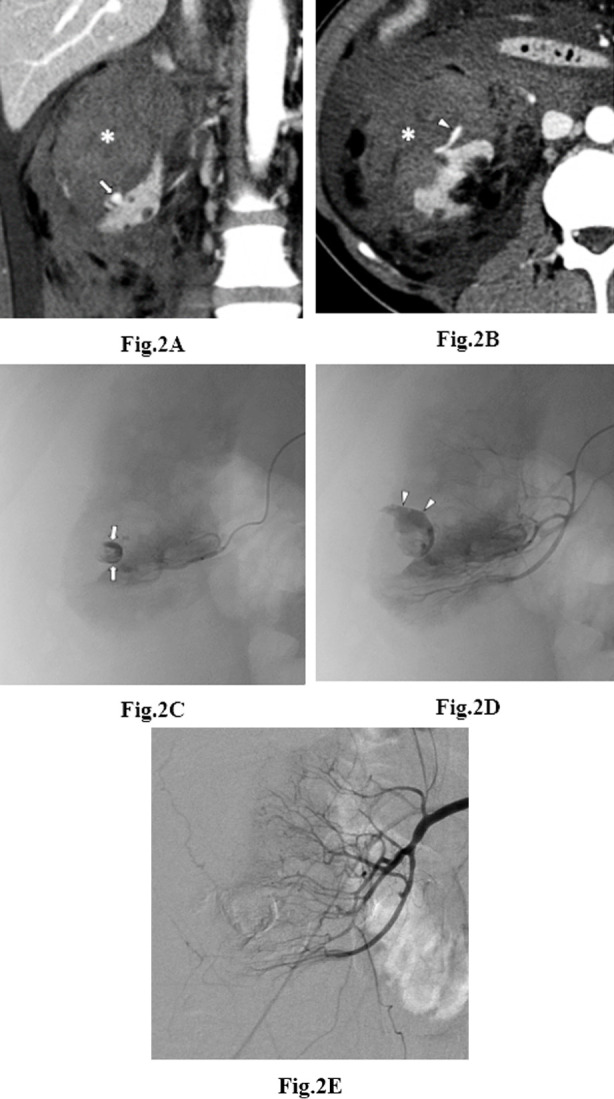
A 32-year-old woman (no. 8) with contrast extravasation. a, b Coronal (a) and axial (b) contrast-enhanced computed tomography scans show a nodular shape (arrow) and subsequent partial spread-out (arrowhead) of the contrast agent, suggesting hemorrhagic cyst rupture at the right renal cortex lower pole with a large subcapsular hematoma (asterisks). c, d Right renal selective angiograms showing contrast agent (arrows in c) confined to the renal cyst and contrast extravasation (arrowheads in d) beyond the cyst boundary. e Right renal angiogram after embolization with n-butyl cyanoacrylate showing partial embolization of the right renal artery.

Angiography was performed in all patients within 40 h after the CT scan (mean, 10.6 hour; range, 2–40 h). Angiography showed active bleeding in 14 patients (78%), suspicious bleeding in one (5%), and no bleeding in three (17%). Active bleeding was contrast extravasation (n=6; Fig. 2), pseudoaneurysm (n=3), and both (n=5). Seven patients (39%) showed multiple bleeding foci. Prophylactic embolization was performed in one patient with suspicious bleeding focus and three patients without angiographic bleeding. A single embolic agent and combination of embolic agents were used in 8 and 10 patients, respectively. The most frequently used embolic agents were NBCA (n=3) and PVA (n=3) for single embolic agents and coils/PVA (n=4), GSP/PVA (n=2), and coils/GSP (n=2) for a combination of embolic agents. Total and partial embolization was performed in 14 (78%) and 4 (22%) cases, respectively.

Technical and clinical success rates were 100% and 94% (17/18), respectively. Clinical failure occurred in one patient (no. 14), with a decreased hemoglobin level of 0.6 g/dL despite blood transfusion and increased hemorrhagic cyst diameter with residual contrast extravasation on 2-day follow-up CT after total renal artery embolization. A surgical nephrectomy was performed on the same day.

There were no major complications. Post-embolization syndrome developed in one patient (no. 3): he had mild abdominal pain, fever, leukocytosis, and an elevated C-reactive protein within 24 hour after embolization, which subsided after 48 h, and there was no evidence of infection. All patients underwent hemodialysis or peritoneal dialysis within 4 days after the procedure. CIN was noted in five patients (nos. 2, 13, 15, 16, and 17; 28%). The elevated sCr level had returned to baseline at the time of discharge in all patients. The mean follow-up period was 981.6±769.4 days (range, 18–2,559 days). Hematoma liquefied or disappeared in patients with follow-up CT over 6 months, and no evidence of RCC in the ruptured kidney was noted, indicating the absence of tumor bleeding.

## DISCUSSION

In the present study, the technical success rate was in the range of 80–100% reported in patients with renal hemorrhage in previous studies.^3^,^11^ The main cause of technical failure in the previous studies was difficulty performing super-selective embolization of the bleeding artery to minimize renal parenchymal damage as much as possible. In this study, total embolization was performed in the majority of patients, which probably led to the high technical success rate. Patients with chronic kidney disease have small, irregular renal arteries. Furthermore, the arterial diameter is prone to be narrowed in the shock condition and can be spastic to catheter or guidewire manipulation,^3^ making super-selective embolization difficult.

The high proportion of multiple bleeding on angiography was another reason for total embolization. Several patients showed multiple active bleeding along the renal cortex indicative of cortical hemorrhage. Cortical hemorrhage is a rare complication of subcapsular hematoma, especially in chronic kidney disease, because an atrophied and scarred cortex may cause renal capsules to be less attached to the parenchyma, which facilitates the development of subcapsular hematoma.^12^ It is hypothesized that in patients with ACKD, ruptured hemorrhagic cysts lead to relatively rapid subcapsular hematoma, causes tearing of the cortical arteries and increased bleeding severity. Indeed, in four cases, the hemoperitoneum probably originated from massive retroperitoneal hemorrhage. Furthermore, unruptured hemorrhagic cysts were observed as pseudoaneurysms in several patients, with potential rebleeding risk requiring treatment. Patients have end-stage kidneys; thus, even with total embolization, the urine formation function does not worsen. Rather, it can prevent further renal bleeding.

In this study, various embolic agents, alone or a combination of embolic agents, were very effective in hemostasis. Except for one case where GSP alone was used, permanent embolic agents were used, and it appears to have contributed to high clinical success. In addition to the coils, PVA, and NBCA used in this study, vascular plugs or Onyx can be used to perform successful renal artery embolization.^13^,^14^ Perhaps because of the reduced renal artery diameter, embolization is relatively fast and can be effective. However, caution is required not to reflux the embolic agent into the aorta since patients with chronic kidney disease have minimal renal blood flow.^15^

The clinical success rate was also high. Despite no obvious active bleeding on angiography, prophylactic embolization contributed to the high clinical success rate. Clinical failure occurred in one patient despite technically successful total embolization. The possible cause seems to be collateral flow from other non-embolized arteries, such as the capsular or lumbar arteries.

There were no cases of devastating complication or mortality in our study. Another study reported a mortality rate of 38% with surgical nephrectomy for spontaneous hemorrhage in patients with ACKD.^16^ Mild post-embolization syndrome developed only in one patient, probably due to acute renal infarction. Generally, the end-stage kidney is less likely to experience infarction due to the small volume of the renal parenchyma.^17^

The risk of CIN increases when contrast agents are injected into the artery rather than into the vein^18^ and closer to the renal artery during aortography.^19^ Chronic kidney disease is the most significant risk factor for CIN, the incidence of which was 38% in patients with renal disease after coronary angiography.^20^ For the aforementioned reasons, the possibility of CIN is high in patients with ACKD undergoing renal artery embolization. However, sCr elevation is caused not only by CIN but also by the renal flow decrease induced by the embolization itself, exaggerating the incidence of CIN in these patients. There were no cases of long-term complications or renal function deterioration due to CIN in this study.

### Limitation of the study

First, a relatively small number of patients was enrolled. However, the prevalence of this patient group is not high, and the data collection time was 20 years. Second, embolic agents and devices were selected at each operator’s discretion. Therefore, it is difficult to evaluate the effect of embolic agents on renal artery embolization.

## CONCLUSION

In conclusion, renal artery embolization was safe and effective for controlling spontaneous renal hemorrhage in patients with ACKD. Active bleeding was observed in 78% of patients undergoing angiography, and the technical and clinical success rates of embolization reached 100% and 94%, respectively. In most patients, total rather than partial embolization was performed. CIN was found in 28% of the patients, although there were no cases of long-term complications or decreased renal function.

### Author’ Contributions:

***Ji Hoon Shin*** is responsible and accountable for the accuracy or integrity of this work.

***Cheng Shi Chen, Hyemin Ahn, Ji Hoon Shin:*** Study concepts and design and Guarantor of integrity of the entire study.

***Cheng Shi Chen, Hyemin Ahn, Ji Hoon Shin, Alrashidi Ibrahim, Jong Woo Kim, Hai-Liang Li, Hee Ho Chu:*** Literature research, Clinical studies, Experimental studies/data analysis and Manuscript editing.

***Cheng Shi Chen, Hyemin Ahn, Ji Hoon Shin, Jong Woo Kim:*** Manuscript preparation.
